# Neural and functional correlates of impaired reading ability in schizophrenia

**DOI:** 10.1038/s41598-019-52669-6

**Published:** 2019-11-05

**Authors:** Clément Dondé, Antigona Martinez, Pejman Sehatpour, Gaurav H. Patel, Rebecca Kraut, Joshua T. Kantrowitz, Daniel C. Javitt

**Affiliations:** 1 INSERM, U1028; CNRS, UMR5292; Lyon Neuroscience Research Center, Psychiatric Disorders: from Resistance to Response Team, Lyon, F-69000 France; 2 University Lyon 1, Villeurbanne, F-69000 France; 3 Centre Hospitalier Le Vinatier, Bron, France; 4 Nathan Kline Institute, Orangeburg, NY USA; 5 Dept. of Psychiatry, Columbia University Medical Center/New York State Psychiatric Institute, New York, NY USA; 6 Ferkauf Graduate School of Psychology, Yeshiva University, Bronx, NY USA

**Keywords:** Language, Neurodevelopmental disorders

## Abstract

Deficits in early auditory processing (EAP) are a core component of schizophrenia (SZ) and contribute significantly to impaired overall function. Here, we evaluate the potential contributions of EAP-related impairments in reading to functional capacity and outcome, relative to effects of auditory social cognitive and general neurocognitive dysfunction. Participants included 30-SZ and 28-controls of similar age, sex, and educational achievement. EAP was assessed using an auditory working memory (tone-matching) task. Phonological processing and reading Fluency were assessed using the Comprehensive Test of Phonological Processing and Woodcock-Johnson reading batteries, respectively. Auditory-related social cognition was assessed using measures of emotion/sarcasm recognition. Functional capacity and outcome were assessed using the UCSD Performance-based Skills Assessment and Specific Level of Functioning scale, respectively. fMRI resting-state functional-connectivity (rsFC) was used to evaluate potential underlying substrates. As predicted, SZ patients showed significant and interrelated deficits in both phonological processing (*d* = 0.74, *p* = 0.009) and reading fluency (*d* = 1.24, *p* < 0.00005). By contrast, single word reading (*d* = 0.35, *p* = 0.31) was intact. In SZ, deficits in EAP and phonological reading ability significantly predicted reduced functional capacity, but not functional outcome. By contrast, deficits in reading fluency significantly predicted impairments in both functional capacity and functional outcome. Moreover, deficits in reading fluency correlated with rsFC alterations among auditory thalamus, early auditory and auditory association regions. These findings indicate significant contributions of EAP deficits and functional connectivity changes in subcortical and early auditory regions to reductions in reading fluency, and of impaired reading ability to impaired functional outcome in SZ.

## Introduction

Deficits in early auditory processing (EAP) are a core component of schizophrenia (SZ) and contribute significantly to impaired functional outcome^1–5^. To date, however, the pathways leading from impaired EAP to impaired functional outcome remain under investigation. We^6,7^ and others^8^ have previously demonstrated impaired phonological reading ability in SZ related to impaired EAP, but the relationship of these deficits to functional impairments has not previously been evaluated.

In a prior study^7^, we evaluated several potential batteries for reading assessment in parallel, and observed greatest effect sizes for two specific tests – alternate (non-word) phonological awareness (APA) on the Comprehensive Test of Phonological Processing-2 (CTOPP)^9^, and reading fluency on the Woodcock-Johnson III Tests of Achievement (WJ)^10^.

The CTOPP-APA assesses the ability to sound-out and manipulate non-words phonemes of non-words (e.g. segment or blend phonemes of non-words) and thus addresses low-level phonological mechanics of reading. By focusing on non-words relative to words, the APA domain minimizes the degree to which associations learned prior to illness onset might influence performance. As opposed to their difficulty in manipulating phonemes, SZ patients show intact performance on other components of the CTOPP battery such as Phonological Memory^7^.

The WJ-fluency test assesses the ability to read simple sentences quickly and then to respond correctly to simple true/false questions. As opposed to CTOPP-APA, reading of connected passages of text as in the WJ-fluency test requires visual as well as phonological reading skills. We^6,11^ and others^8^ have documented significant contributions of early visual processing impairments (e.g. contrast sensitivity, parafoveal processing) to reading impairments in SZ. The CTOPP-APA and WJ-Fluency tests thus provide complementary information regarding reading-related processing skills in SZ. Furthermore, these subsets do not require a trained rater, and so can be obtained within routine clinical settings.

Here, we evaluated CTOPP-APA and WJ-Fluency scores relative to both EAP and functional capacity/outcome. EAP deficits were assessed using a previously described delayed tone-matching paradigm^2^. In our prior study involving over 500 subjects, we observed that EAP deficits were bimodally distributed in SZ, with one group of patients – termed SZ-EAP + – showing preserved abilities (mean 87.8 ± 6.8%) that overlapped those of HC (mean 84.9 ± 9.6%)) and a second group of patients –termed SZ-EAP- — showing marked impairments (mean 64.1 ± 8.4%) relative to controls^12^. The critical threshold separating the EAP + and EAP- groups was 77.7% correct performance, which was therefore implemented here to define the groups.

We have also previously reported on the deficits in specific aspects of social cognition in SZ, including impaired auditory emotion recognition^13–15^ and sarcasm^16–18^ detection, but have also not previously evaluated the degree to which these deficits contribute to impaired functional outcome. As a secondary analysis, we evaluated the potential contributions of both reading ability and social cognitive impairment, along with general neurocognitive abilities as measured by the MATRICS Battery, to impaired outcome in SZ.

Functional capacity and outcome were assessed using the UCSD Performance-based Skills Assessment (UPSA)^19^ and the Specific Level of Functioning (SLOF)^20^ tests, respectively. The UPSA is test-based and assesses the ability of individuals to perform a number of tasks relevant to everyday function such as managing money, comprehending written information and planning activities^19^. The SLOF by contrast is interview based and assesses specific aspects of actual functional abilities such as personal care, interpersonal communication and working skills^20^. Both tests are sensitive to functional outcome impairments in SZ and have increasingly been adopted as “gold standard” measures for their respective domains.

Finally, we used fMRI resting state functional connectivity (rsFC) to evaluate potential substrates of impaired reading ability within the early auditory system. rsFC is thought to reflect some combination of anatomical connectivity and functional coupling between areas and can thus reveal potential deficits related to behavioural deficits^21,22^. We have recently observed that tone-matching deficits in SZ reflect impaired rsFC between subcortical and cortical auditory structures^23^. Here, we performed similar analyses relative to reading ability.

Our study was designed with the following aims: (i) confirm our prior observations of significant deficits in both phonological and visual components of reading in SZ using well-validated tasks, (ii) evaluate the relationship between reading deficits and functional capacity/outcome relative to other potential mediators, (iii) evaluate the relationship between reading deficits and EAP measured based on tone-matching ability, and (iv) evaluate potential underlying neural substrates using rsFC-fMRI.

## Results

### Social demographic, neurocognition and EAP differences between groups

No significant differences were observed between groups for age, sex, handedness, achieved education or parental socioeconomic status (SES) (Table 1). As expected, patients showed reduced individual SES relative to controls (*p* < 0.05).

**Table 1:** Reading, auditory and cognitive abilities across groups. Data is presented as mean ± std. dev.

Variable	Group		Statistics			
	HC (*N* = 28)	SZ (*N* = 30)	Test	Stat	p-value	Effect Size
**Demographics**						
Age (years)	37.2 ± 10.2	39.4 ± 11.2	*t*	0.78	—	
Sex (F/M)	6/24	11/21	*χ* ^2^	1.61	—	
Ethnicity (%)						
Hispanic latino	10.7%	20%	*χ* ^2^	0.95	—	
White Caucasian	32.1%	40%	*χ* ^2^	0.39	—	
Black	53.6%	33.3%	*χ* ^2^	2.42	—	
Asian	3.6%	6.7%	*χ* ^2^	0.28	—	
Hand preference (L/F)	3/25	1/29	*χ* ^2^	1.22	—	
Highest grade achieved	14.9 ± 2.0	14.1 ± 2.5	*z*	0.82	—	
Participant SES	35.0 ± 13.8	26.5 ± 10.0	***z***	**1.40**	**< 0.05**	**0.37**
Parents SES	45.4 ± 13.0	41.7 ± 14.9	*t*	1.00	—	
Antipsychotic medication	—	Atypical: 25/30 Typical: 3/30 Combination: 2/30				
Chlorpromazine equivalent^46–48^	—	528.5 ± 735.9	—	—	—	
**Neurocognition** (MCCB T-scores)						
**Total**	—	40.7 ± 7.7	—	—	—	
Processing speed	—	39.1 ± 10.8	—	—	—	
Attention/Vigilance	—	43.9 ± 13.3	—	—	—	
Working Memory	—	40.9 ± 11.5	—	—	—	
Verbal Learning	—	41.0 ± 7.2	—	—	—	
Visual Learning	—	37.5 ± 12.5	—	—	—	
Reasoning/Problem Solving	—	41.9 ± 11.3	—	—	—	
**Social cognition** (% Total)						
Auditory emotion recognition	67.6 ± 11.8	57.8 ± 13.9	***t***	**2.89**	**0.005**	**0.76**
Sarcasm	82.7 ± 13.0	75.1 ± 14.6	***t***	**2.09**	**0.041**	**0.55**
**Early Auditory Processing**						
Tone-matching (% Total)	83.2 ± 11.7	82.3 ± 11.7	*t*	0.29	—	
**Functioning**						
SLOF (mean)	—	4.7 ± 0.3	—	—	—	
UPSA (scaled score)	—	77.5 ± 11.0	—	—	—	

Although MATRICS Consensus Cognitive Battery (MCCB) scores were not obtained in controls, SZ patients showed an expected deficit of ~1 std. dev. relative to published norms across neurocognition domains (Table 1). EAP was assessed using a previously described tone-matching task. Consistent with recruitment site (outpatient setting), no overall deficits in EAP were observed^23,24^.

### Reading and social cognition differences between groups

**Figure 1:**
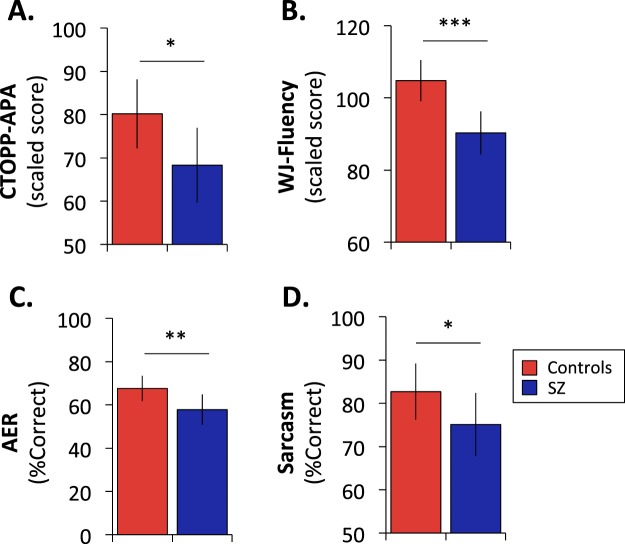
Behavioral assessment of reading (**A**. CTOPP-APA, **B**. WJ-Fluency) and auditory-related social cognition (**C**. Auditory Emotion recognition, D. Sarcasm). Data are mean ± std. dev. ***p < 0.0005, **p < 0.005, *p < 0.05

As expected, SZ patients showed significant moderate-large effect-size deficits in CTOPP-APA (*t*_*(1,56)*_ = 2.70, *p* = 0.009, *d* = 0.74) (Fig. 1A) and CTOPP-phonological awareness (*t*_*(1,35)*_ = 2.09, *p* = 0.04, *d* = 0.69), although CTOPP-phonological memory was intact (*t*_*(1,35)*_ = 1.04, *p* > 0.05) (Table 2).

**Table 2:** Reading, auditory and cognitive abilities across groups. Data is presented as mean ± std. dev. *data available for only a subsample of participants (17 HC, 20 SZ).

Variable	Group		Statistics			
	HC (*N* = 28)	SZ (*N* = 30)	test	stat	p-value	effect size
**Comprehensive Test of Phonological Processing II** (scaled scores)						
Alternate phonological awareness	80.2 ± 16.0	68.3 ± 17.3	*t*	2.70	**0.009**	**0.74**
Phonological awareness*	88.6 ± 15.4	77.0 ± 17.9	*t*	2.09	**0.04**	**0.69**
Phonological memory*	97.9 ± 12.3	91.4 ± 23.0	*t*	1.04	0.31	0.35
**Woodcock-Johnson III Tests of Achievement** (scaled scores)						
Basic reading	99.9 ± 11.6	96.8 ± 10.6	*t*	1.06	0.31	0.35
Reading fluency	104.8 ± 11.4	90.3 ± 12.0	*t*	4.70	**<0.00005**	**1.24**
Reading comprehension	99.9 ± 9.5	94.1 ± 8.4	*t*	2.47	**0.02**	**0.65**

On the WJ, no deficits were observed in Basic Reading, which reflects ability to identify isolated words (*t*_*(1,56)*_ = 1.06, *p* = 0.28) (Table 2). By contrast, deficits in WJ-Fluency (*t*_*(1,56)*_ = 4.70, *p* < 0.00005, *d* = 1.24) (Fig. 1B) and WJ-Comprehension (*t*_*(1,56)*_ = 2.47, *p* = 0.02, *d* = 0.65) (Table 2) were significant. An rmANOVA comparing WJ-Fluency to both WJ-Basic Reading and WJ-Comprehension showed both a highly significant main effect of group (*F*_(1,55)_ = 12.8, *p* < 0.00001) and a highly significant group X test interaction (*F*_(2,54)_ = 5.64, *p* = 0.006), with significant differential deficits of Fluency vs. both Basic Reading (*F*_(1,55)_ = 8.43, *p* = 0.005) and Comprehension (*F*_(1,55)_ = 10.9, p = 0.002).

*Social cognition:* As expected, SZ patients showed significant AER (*t*_*(1,56)*_ = 2.89, *p* = 0.005, Fig. 1C) and Sarcasm (*t*_*(1,56)*_ = 2.09, *p* = 0.041, Fig. 1D) deficits relative to HC.

### Impact of early auditory processing and reading on functional outcome

**Figure 2:**
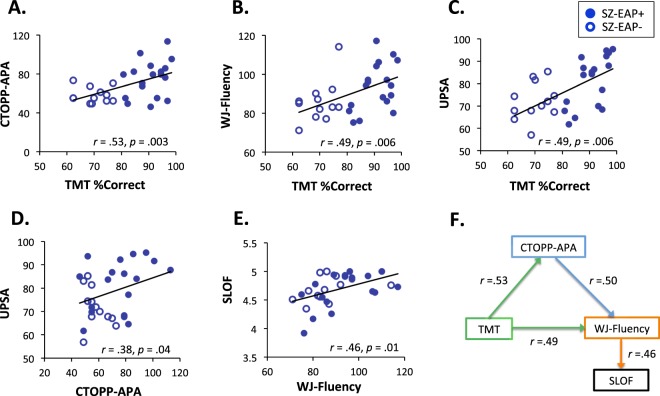
Relationships (Pearson’s *r*) between reading, tone-matching and functioning measures in the SZ group. SZ-EAP- and SZ-EAP + refers to subtypes of patients that are significantly different according to tone-matching performance, with SZ-EAP- defined as below a threshold of 77.7% correct responses^12^.

Tone matching correlated significantly with CTOPP-APA and WJ-Fluency both across groups covaried for group membership (CTOPP-APA: *r*_*p*_ = 0.59, *p* < 0.0001; WJ-Fluency: *r*_*p*_ = 0.29, *p* = 0.029) and in SZ subjects alone (Fig. 2A,B). Tone-matching performance also correlated significantly with UPSA (Fig. 2C), but not SLOF scores within patients, and with multiple neurocognitive domains of the MCCB (Processing Speed: *r* = 0.50, *p* = 0.005; Attention/Vigilance: *r* = 0.39, *p* = 0.03; Working memory: *r* = 0.54, *p* = 0.002; Verbal Learning: *r* = 0.59, *p* = 0.001; Total: *r* = 0.59, *p* = 0.001).

CTOPP-APA scores correlated significantly with UPSA (Fig. 2D) but not SLOF (*r* = 0.23, *p* = 0.23). WJ-Fluency scores correlated with both UPSA (*r* = 0.49, *p* = 0.006) and SLOF (Fig. 2E). By contrast to reading scores, AER correlated marginally with the UPSA (*r* = 0.36, *p* = 0.052) and did not correlate to SLOF.

### Interrelationship among measures

Hierarchical regressions were performed to evaluate the potential role of reading, auditory-related cognition, and other neurocognitive domains on functional capacity/outcome in the SZ group (Table 3).

**Table 3:** Partial correlation amongst reading, cognition and functional capacity/outcome in the SZ group. AER: Auditory Emotion Recognition; CTOPP-APA: Comprehensive Test of Phonological Processing II – Alternate Phonological Awareness; MCCB: MATRICS Consensus Cognitive Battery; WJ: Woodcock-Johnson III Tests of Achievement; SLOF: Specific Level of Functioning; UCSD: Performance-based Skills Assessment. *r*_p_ = partial correlation *r*.

Variables		Covariable(s)	*r*	p-value
UPSA	Tone-matching task	None	***r* =** 0.**49**	**0.006**
		Combined reading (CTOPP-APA, WJ-Fluency) and auditory-related social cognition (AER, Sarcasm)	***r***_**p**_** =** 0.**43**	**0.03**
		Neurocognition (overall MCCB)	***r***_**p**_** =** 0.**41**	**0.03**
		Auditory-related neurocognition (MCCB Working memory, MCCB Verbal learning)	*r*_p_ = 0.36	0.06
UPSA	MCCB Working memory	None	***r* =** 0.**71**	**0.00001**
		CTOPP-APA	***r***_**p**_** =** 0.**66**	**0.0001**
SLOF	WJ-Fluency	None	***r* =** 0.**46**	**0.01**
		Neurocognition (overall MCCB)	***r***_**p**_** =** 0.**42**	**0.024**
		UPSA	***r***_**p**_** =** 0.**40**	**0.03**
SLOF	WJ-Comprehension	None	***r* =** 0.**37**	**0.045**
		WJ-Fluency	***r***_**p**_** =** 0.**37**	**0.045**
SLOF	Neurocognition (overall MCCB)	None	*r* = 0.23	0.23
		WJ-Fluency	*r*_p_ = −0.047	0.81
SLOF	MCCB Working memory	None	***r* =** 0.**42**	**0.02**
		WJ-Fluency	*r*_p_ = 0.24	0.21

For UPSA-defined functional capacity, the relationship with tone-matching remained significant following covariation for reading (CTOPP-APA, WJ-Fluency) and AER/Sarcasm measures (*r*_*p*_ = 0.43, *p* = 0.03). The correlation also remained significant following control for overall MCCB score (*r*_*p*_ = 0.41, *p* = 0.03). However, the correlation between tone-matching and UPSA was no longer significant (*r*_*p*_ = 0.36, *p* = 0.06) once auditory-dependent domains of the MCCB (Working memory, Verbal learning) were considered individually.

Working memory scores also correlated highly with UPSA scores (*r* = 0.71, *p* = 0.00001). Combined Reading (WJ-Fluency) and Working memory scores accounted for > 50% of the variance in UPSA (R² = 0.57, *p* < 0.0001).

For SLOF-defined functional outcome, the relationship with tone-matching (*r* = 0.46, *p* = 0.01) remained significant even following covariation for MCCB total score (*r*_*p*_ = 0.42, *p* = 0.024) or UPSA (*r*_*p*_ = 0.40, *p* = 0.03). Once WJ-Fluency was considered, there were no significant correlations of MCCB total score (*r*_*p*_ = −0.047, *p* = 0.81) or Working memory specifically (*r*_*p*_ = 0.24, *p* = 0.21) to SLOF. By contrast, the correlation between WJ-Comprehension and SLOF remained significant following covariation for WJ-Fluency (*r* = 0.37, *p* = 0.045). When both WJ measures (Fluency and Comprehension) were entered into a stepwise regression, only the correlation with WJ-Fluency remained significant.

### rsFC-MRI

**Figure 3:**
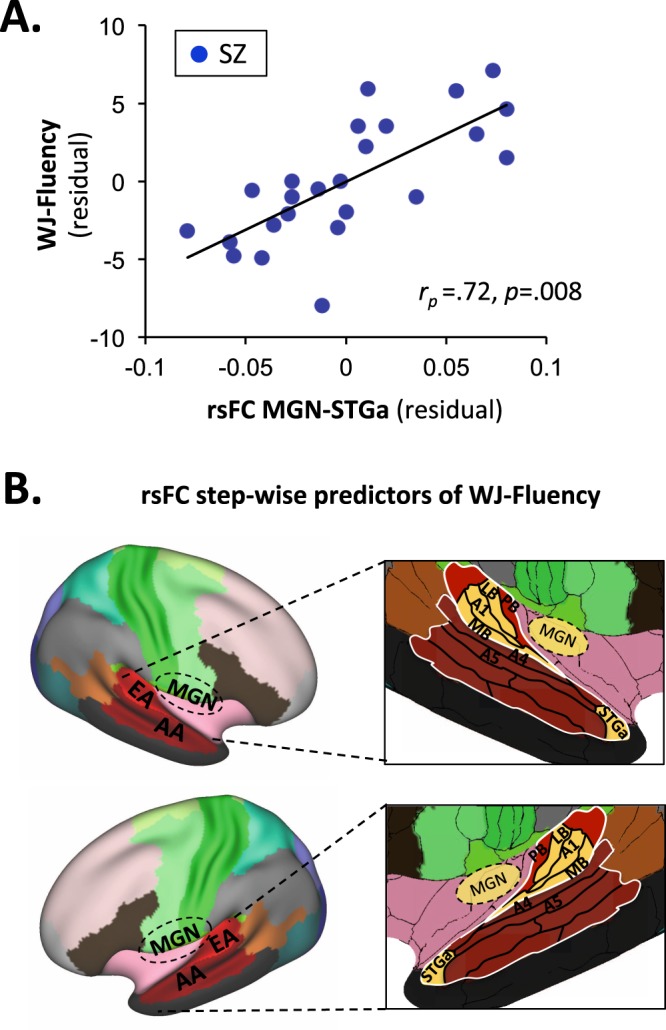
Relationships between reading and rsFC in the SZ group. Regions are bilateral medial geniculate nucleus (MGN), Early Auditory region (EA) and Associative Auditory region (AA). Specific-language related parcels for EA include primary auditory cortex (A1), Medial belt (MB), Lateral belt (LB), and Parabelt (PB). Specific-language related parcels for AA include A4, A5 and anterior Superior Temporal Gyrus (STGa). (**A**) Correlation between WJ-Fluency and rsFC between MGN and STGa. (**B**) Parcels involved in step-wise rsFC predictors of WJ-Fluency. Significant parcels are highlighted in yellow.

In order to assess underlying neural auditory mechanisms, we evaluated pairwise rsFC between bilateral MGN and A1, and language-related regions in EA (MBelt, LBelt, PBelt), and AA (A4, A5, STGa) within patients, using a prespecified multimodal imaging parcellation scheme^25^ (Fig. 3A).

For CTOPP-APA, the overall regression was not significant (*F*_(13,10)_ = 1.79, *p* = 0.17). Nevertheless, significant partial correlations were observed for MGN-MBelt (*r*_p_ = 0.70, *p* = 0.008) and MGN-PBelt (*r*_p_ = −0.59, *p* = 0.034).

For WJ-Fluency, the overall regression was highly significant (*F*_(13,10)_ = 13.8, *p* < 0.0001) and accounted for > 80% of the variance (adj R² = 0.88). Significant independent correlations were observed for MGN-MBelt (*r*_p_ = 0.93, *p* < 0.0001), MGN-PBelt (*r*_p_ = −0.87, *p* < 0.0001), MGN-STGa (*r*_p_ = 0.72, p = 0.008, Fig. 3A), A1-PBelt (*r*_p_ = −0.72, *p* = 0.009) and A1-STGa (*r*_p_ = 0.86, *p* < 0.0001). The combination of MGN-MBelt (*r*_p_ = 0.55, *p* = 0.008), MGN-STGa (*r*_p_ = 0.65, *p* = 0.001) and A1-LBelt (*r*_p_ = −0.68, *p* = 0.001) accounted for ~50% of the variance (adj. R² = 0.48, *F*_(3,20)_ = 8.07, *p* = 0.001) (Fig. 3B).

## Discussion

EAP deficits^2,3,26^ and their relationship with functional outcome^4,27^ are increasingly documented in SZ, but potential mediators remain under investigation. Over recent years, we^6,7^ and others^8^ have also documented reading deficits in SZ related in part to EAP impairments and identified two reading tests – CTOPP-APA and WJ-Fluency – that are particularly sensitive to deficits in SZ. Here we replicated our prior findings of reading deficits in SZ and show that it is present even when patients are compared to controls with similar educational achievement.

We also investigated the relationship of reading impairments to functional outcome relative to contributions of other auditory-related and general neurocognitive impairments. Primary findings are that deficits in Reading Fluency along with Auditory Working Memory mediate the relationship between EAP deficits and reduced functional capacity assessed using the UPSA, and that deficits in Reading Fluency also contribute statistically to SLOF-defined functional outcome over and above effects of deficits in auditory social cognition and overall neurocognition (MCCB). These findings confirm and extend prior results regarding EAP^1–3^, AER/sarcasm^16–18,28^ and reading^6–8^ deficits in SZ.

EAP deficits were first demonstrated more than 40 years ago^29^, but have been increasingly studied over the last 2 decades^2,3,26^. Patients as a group show significant EAP deficits^2^ that are bimodally distributed, suggesting the existence of discrete etiological subgroups^23^. EAP deficits are detected behaviourally using tone-matching task as in the present study and neurophysiologically using measures such as mismatch negativity (MMN)^5,24,30,31^, which reflect preattentive detection of stimulus change within supratemporal auditory cortex.

Prior studies have demonstrated significant correlations between MMN deficits and overall functional outcome mediated in part through neurocognition as measured using MCCB^4^. Similarly, behaviourally defined EAP deficits have been shown to correlate significantly to deficits in auditory social cognition including AER and Sarcasm^13,15,32^. Most recently, it has been reported improvement in EAP during auditory training intercorrelates with improvement in Verbal Learning^33^ or Attention/Vigilance^5^, consistent with results of this study.

With regard to reading, it has been appreciated for decades that patients show impairments in reading comprehension^8^. However more basic reading skills such as phonological processing and reading fluency have been investigated to a lesser extent^7^. In a prior head-to-head comparison of multiple normed reading batteries, we suggested that two specific reading measures, CTOPP-APA and WJ-Fluency, are particularly sensitive to deficits in SZ and, in addition, are well-suited for routine assessment in clinical setting^7^. Here, we again observed a differential deficit in reading fluency compared to comprehension. Thus, the present study confirms the presence of large effect size deficits in specific reading domains in SZ, even compared to controls with similar educational achievement.

We also evaluated contributions of reading, relative to other auditory-related functions and general neurocognition, as a potential moderator of the relationship between EAP and outcome. As predicted, deficits in tone-matching correlated significantly with UPSA scores, as did reading ability and auditory working memory. The combination of Reading Fluency and Working Memory accounted for ~50% of the variance in UPSA. Once these measures were taken into account, other auditory or cognitive domains were without further effect.

Tone-matching scores did not directly predict SLOF scores in SZ. However, both tone-matching and phonological processing correlated significantly with impaired WJ-Fluency, which in turn correlated significantly with SLOF score. The correlation remained significant even following control for MCCB and UPSA. Notably, deficits in Reading Fluency showed stronger correlations to functional outcome than did deficits in Comprehension, emphasizing the importance of low-level sensory correlates of impaired outcome in SZ.

These results support prior findings showing a significant relationship between reading deficits and outcome in CHR^7,34^. In that study, deficits in both MMN and reading significantly predicted impaired role function even in individuals with intact premorbid IQ as detected by single-word reading. As in the present study, reading deficits correlated significantly with impaired EAP, and correlations to outcome were more significant with Fluency than Comprehension measures.

Our results show that SZ patients had intact single-word recognition (Basic reading), suggesting relatively intact premorbid function. Fluency, reflecting present reading ability for connected text, was significantly impaired relative to Basic reading. Overall, these findings support a model in which SZ patients, as a group, experience a significant decline in reading ability from a higher premorbid level, and that such deficits occur early in the course of the illness and may predate illness onset^7^. Given the link to functional outcome both in CHR and established SZ, these findings underscore the need for reading evaluation early in the course of illness and implementation of appropriate interventions.

The present study also evaluated potential neural substrates underlying impaired reading ability. We have previously shown that EAP deficits in SZ correlate with decreased MGN-STGa connectivity^23^ whereas visual components of reading correlate with connectivity in brain regions related to eye movement control and early visual processing (e.g. inferior frontal gyrus (IFG)-frontal eye field (FEF); superior colliculus (SC)- middle occipital gyrus (MOG))^11^. Here we show additionally that reading fluency deficits in SZ depend on functional connectivity between the MGN, early (EA) and association (AA) cortical regions including parcels A1, LBelt/MBelt and STGa. STGa in particular is known to play a prominent role in phonetic processes and comprehension of speech^35–37^. Thus dysconnectivity between subcortical, EA and AA regions, especially STGa, may contribute significantly to impaired reading in SZ.

*Limitations:* Despite the original findings, several limitations should be acknowledged. First, all subjects were receiving antipsychotic medications. Thus, the potential contribution of medication to the pattern of results cannot be assessed. Nevertheless, no significant correlations were observed with medication dose expressed in chlorpromazine equivalents. Second, although we report correlates of reading disability and specific functional circuits, no imaging was performed during the reading tasks themselves. Future studies are needed to assess potential activation differences in auditory thalamic, EA and AA during reading in Sz. Finally, the sample size is relatively small (n = 28–30/group), and relationships are correlational rather than causal, necessitating replication in larger cohorts and with interventional approaches to evaluate causality.

In sum, SZ is associated with impairments in interpersonal, work, or self-care functions. Here, we demonstrate significant interrelations between EAP deficits, reductions in phonological processing/reading fluency and impaired functional outcome in SZ, over and above effects of general neurocognitive ability. We also replicate the utility of two specific measures – CTOPP-APA and WJ-Fluency – for assessing reading ability within larger clinical SZ populations. Reading deficits were tied to impaired functional connectivity between MGN, EA and AA regions, suggesting potential targets for future remediation- or neurostimulation-based interventions.

## Methods

### Participants

Participants included 30 individuals with a DSM-IV diagnosis of SZ disorder and 28 representative healthy controls. Demographic information is detailed in Table 1. Patients were recruited from outpatient settings associated with Columbia University Medical Center (CUMC). Patient diagnoses were established using the Structured Clinical Interview for DSM-IV (SCID) for SZ.

Controls were recruited by advertisement from the local communities at the recruitment sites, as well as from registries of prior study participants. They were screened using the Structured Clinical Interview for DSM-IV to make sure they were free of a current DSM disorder and had no lifetime history of SZ-spectrum disorder and were of similar age and educational status as the SZ participants.

All participants were free of substance dependence within the past six months and substance abuse in the past month (DSM-IV). All denied a history of head injury with loss of consciousness, or other neurological disorders. All participants provided written, informed consent. The study was approved by the CUMC/NYSPI (New York State Psychiatric Institute) institutional review board/ethics committee.

All methods were performed in accordance with the relevant guidelines and regulations.

### Measurements

#### Reading measures

Assessment batteries included the alternate phonological awareness (CTOPP-APA) and phonological memory scores of the CTOPP battery^9^, and the Fluency (WJ-Fluency), Basic and Broad Reading scales of the WJ battery^10^. WJ-Fluency is based upon timed reading of connected text consisting of short sentences followed by simple questions regarding content. WJ-Comprehension is based upon ability to understand spoken passages. By contrast, Basic Reading measures they ability to identify single words, while Broad Reading, encompasses measures of Fluency and Comprehension.

Both the CTOPP and WJ measures are standardized to a distribution mean of 100 and std. dev. of 15.

#### Clinical measures

*Neurocognition*: The first six modules of the MCCB (Speed of processing, Attention/ vigilance, Working memory, Verbal learning, Visual learning, Reasoning/ problem solving) were administered to patients. The primary measures were T-scores for each module as well as overall.

*Social cognition:* Auditory-related social cognition was assessed using the Auditory Emotion Recognition (AER) and sarcasm “attitudinal prosody” (Sarcasm) tests as previously described^18,38^. The AER test consists of 32 stimuli in which a neutral sentence (“It’s 11O’clock”) is read in happy, sad, angry, fearful and no emotion (neutral) tone of voice. Sarcasm detection was assessed using the attitudinal subtest of the Aprosodia Battery^39^. This test consists of 10 semantically neutral sentences uttered in either a sincere or sarcastic tone of voice. For both AER and Sarcasm, the primary measure consists of % correct responses across all stimuli.

*Symptoms*: Symptoms were assessed in SZ participants using a prespecified 5-factor solution of the Positive and Negative Symptoms Scale (PANSS)^40^, encompassing separate positive, negative, cognitive, excitement/hostility, and anxiety/depression factors. Mean (±std. dev.) scores were 47.3 ± 14.1 (Total), 10.9 ± 4.6 (Positive factor), 12.8 ± 4.2 (Negative factor), 8.5 ± 2.8 (Cognitive factor), 10.5 ± 4.5 (Depression/anxiety factor) and 6.3 ± 2.5 (Excitement/hostility factor).

*Functional status:* Functional capacity was measured with the UCSD Performance-based Skills Assessment (UPSA) task. Functional outcome was assessed with the Social level of functioning (SLOF) battery.

#### Early auditory processing measure

*Tone-matching task:* Integrity of EAP was assessed using a simple tone-matching paradigm as described previously^1,2,17^. This task presents subjects pairs of 100-ms tones in series, with 500-ms intertone interval. Within each pair, tones are either identical or differ in frequency by specified amounts in each block (Δf = 2.5, 5, 10, 20 or 50%). Tones are derived from 3 reference frequencies (500, 1000, and 2000 Hz) to avoid learning effects. In all, the test consists of 5 blocks of 26 pairs of tones. Participants listen to the tone pairs and then indicate either verbally or by button press whether the two tones were the same or different. Percent correct responses are calculated across Δf levels.

#### rsFC-MRI measures

*Acquisition*: Resting state fMRI scans were obtained from 24 SZ patients and 25 controls and used to calculate pairwise rsFC values per subject. Data were acquired on a GE MR750 3.0T whole-body scanner located in the MRI Research Unit at New York State Psychiatric Institute/CUMC using a multiband SMS-EPI sequence (courtesy of the Center for Cognitive and Neurobiological Imaging, Stanford University, http://cni.stanford.edu). 3–6 BOLD runs (median 4 BOLD runs) were collected for each subject; each BOLD run was 5.5 minutes in length. On the scanner bed, participants were asked to fixate on a white dot in the center of a black screen for the BOLD run. MR-related sounds were minimized with earplugs and potential head movements were stabilized with cushioning.

*Processing:* MRI data were pre-processed with the Human Connectome Project (HCP) pipelines v3.4^41^. The HCP pipelines first processed the anatomy images to create a cortical surface model for each individual aligned to the HCP fs_LR 32k atlas, then for the functional runs performed movement correction, distortion correction, and atlas alignment in a single resampling step, and lastly projected the functional data to an atlas cortical surface through the individual’s cortical surface model, creating a Connectivity Informatics Technology Initiative (CIFTI) file for each BOLD run that only contains the data from the cortical and the subcortical grey matter (“gray-ordinates” as opposed to voxels).

Structural and functional images were aligned in a volume space of the Montreal Neurological Institute (MNI152) atlas and on the surface Conte69/fs LR 32k atlas created by the HCP pipeline^41–43^. Additional post-processing procedures were performed to minimize confounding artifacts adapted from the recommendations by Power *et al*.^44^. This included removal by regression of signals related to head motion, white matter/CSF/global brain signals and their derivatives, censoring of MR frames with a Framewise Displacement > 0.2 mm and replacement by interpolation, and temporal bandpassing of data between 0.0005–0.588 Hz.

*Analysis*: Correlations between rsFC and reading measures were assessed using the rsFC between predefined language-network related parcels from a recently validated multimodal-imaging based cortical atlas^25^. Timecourses were averaged across gray-ordinates within each parcel, and then used to calculate parcel-parcel correlations (Pearson’s correlation), which was normalized by Fisher-z transformation. Within the Early Auditory (EA) region, these included primary auditory cortex (A1), Lateral belt (LB), Medial belt (MB), and Parabelt (PB). Within the Auditory Association (AA) region, these included A4, A5 and anterior superior temporal gyrus (STGa). An additional ROI was delineated in medial geniculate nucleus (MGN) as defined in the Talairach atlas^45^.

### Statistical analyses

All statistics were carried out using SPSS and R software packages for Mac. For all tests, significance was set at *p* < 0.05. Values in text represent mean ± std. dev. unless otherwise specified. Effect sizes were calculated and interpreted using the Cohen’s d statistic.

Profiles across reading skills, neurocognitive modules, functioning domains and rsFC-fMRI patterns were assessed using repeated measures ANOVA with simple contrasts across measures as appropriate. Effects of potential covariates were assessed using ANCOVA. Group socio-demographic and testing measures performances were compared using independent-sample mean comparison tests for continuous variables, and χ² tests for categorical data.

Correlational analyses for individual potential predictors were conducted using Pearson parametric testing (*r*). Relative contribution of independent predictors was assessed using partial correlation coefficients (*r*_p_) and multiple linear regression models.
